# Proposal for a screening questionnaire for detecting habitual mouth breathing, based on a mouth-breathing habit score

**DOI:** 10.1186/s12903-018-0672-6

**Published:** 2018-12-13

**Authors:** Masahiro Sano, Sayaka Sano, Hiromasa Kato, Ken Arakawa, Masaaki Arai

**Affiliations:** 1 Medical Corporation Chitokukai Family Dental Clinic, 3-17-15, Shimorenjaku, Mitaka-shi, Tokyo, 181-0013 Japan; 2 Kato Dental Clinic, Matsuyama-shi, Ehime Japan; 3 Arakawa Dental Clinic, Urayasu-shi, Chiba Japan; 4 Department of Oral Biomedical Research, Total Health Advisers Co., Chiba-shi, Chiba Japan

**Keywords:** Nasal breathing, Nose breathing, Mouth breathing, Dentistry, HMB score, Asthma, Open mouth posture, Abnormal swallowing

## Abstract

**Background:**

When mouth breathing becomes habitual, it can cause sleep disorders and abnormal maxillofacial growth, thus early detection of habitual mouth breathing is important. We created a questionnaire for early detection of habitual mouth breathing using a score based on a spectrum of factors found to be characteristic of mouth breathers.

**Methods:**

First, a draft 50-question questionnaire was given to 101 random dental clinic patients, classified by dental professionals into habitual mouth breathers (*n* = 28) and nose breathers (*n* = 73). The 10 questions that significantly differentiated mouth and nose breathers (*p* < 0.05) were identified from this questionnaire. These questions, regarding nasal obstruction, open mouth at rest, awareness of mouth breathing, gum swelling and dental staining of the front teeth, bad breath, maxillary protrusion, nasal obstruction in childhood, bottle-feeding, and history of asthma, formed the basis for a second questionnaire. This second survey was completed by another 242 participants, separately classified into mouth breathing (*n* = 26), suspected mouth breathing (*n* = 40), and nose breathing groups (*n* = 176).

**Results:**

Receiver operating characteristic curve analysis of the resulting mouth breathing habit scores, representing the responses to the 10-question survey, showed moderate checklist diagnosability. Sensitivity of cut-off values was 61.5% (specificity 92.0%) for the mouth-breathing group, and 77.5% (specificity 56.3%) for the suspected mouth-breathing group.

Information was also obtained from visual assessment of maxillofacial characteristics. We found that the mouth-breathing and suspected mouth-breathing groups showed significantly high odds ratios for 7 items: discomfort while breathing and increased chin muscle tonus with lip closure, maxillary protrusion, tongue thrust, open mouth at rest, open bite, and childhood asthma. For 94.6% of the nose breathing group, ≥1 of these items applied.

**Conclusions:**

These findings were then used together to create a sample screening form. We believe that screening of this kind can facilitate more accurate diagnosis of habitual mouth breathing and contribute to its early detection.

## Background

Mouth breathing has been reported to cause abnormal facial growth [1–4], attention problems associated with sleep disorders [3, 4], and of reduction in quality of life [5, 6]. In addition, the authors have reported increased oxygen load in the prefrontal cortex when changing from nasal breathing to mouth breathing [7]. In this way, in the field of dentistry, the characteristics of mouth breathing have been investigated mainly for the purpose of discovering the effects of mouth-breathing and identifying habitual mouth breathers.

There are a number of findings reported as characteristic of mouth breathing, but they differ in different reports. For example, there are reports focused on rhinitis and open mouth at rest. Rhinitis is reported as a finding related to mouth breathing in some studies [4, 8–10], but not in others [11, 12]. Open mouth at rest is also found to be characteristic of mouth breathing in some studies [10, 13, 14] but not others, because there are some cases in which the mouth is habitually open without mouth breathing [7, 15, 16]. Mouth breathing is determined by a combination of predisposing factors (i.e., facial type) and precipitating factors (i.e., local factors) [8]. In other words, all of the findings associated with mouth breathing may not apply to a given individual. Accordingly, to identify whether a person is a habitual mouth breather, he or she should be evaluated based not on the presence (or absence) of certain specified characteristics, but on the extent to which the various different characteristics in the spectrum of characteristics associated with mouth breathing apply.

Clinically, visual assessment is most commonly used (97.2%) to identify the characteristic findings of mouth breathing [17]. In visual assessment, the dentist (or other clinician) observes the patient for the presence of factors causing increased breathing resistance, such as adenoid facies, pharynx or palatine tonsil hypertrophy and deviated nasal septum [18]; and observes whether the mouth-breathing route is closed at rest [10, 13, 14]. Facial morphology and the condition of the front teeth and gingiva are also observed. Next in frequency after visual assessment, interviews (87.2%) and respiratory tests (59%) have been used to evaluate for mouth breathing [17]. In interviews, subjects are questioned about symptoms or habits that may induce mouth breathing, such as allergic rhinitis, nasal congestion, snoring, and open mouth during sleeping or resting [11, 17]. Respiratory tests used include a lip seal test, which evaluates whether a subject can keep his or her lips closed; a mirror test, which assesses the extent of clouding on a mirror held below the nose; and a water retention test, which evaluates the ability to hold water in one’s mouth [11].

Namely, the methods and content of evaluation for detecting habitual mouth breathing are left to the discretion of the dentist. There is currently no unified screening method for detecting habitual mouth breathing. Moreover, there is little general awareness of the need for intervention in the case of habitual mouth breathing, and people in general do not know how to identify habitual mouth breathing. For this reason, early detection of habitual mouth breathing is delayed, and people are unlikely to ask their dentist about mouth breathing, or visit a dentist with mouth breathing as their main complaint. Creation of a screening process for detecting habitual mouth breathing without the use of special equipment would thus be useful not only for dentists, enabling them to detect mouth breathing on a uniform scale, but also for the general public, by raising awareness of the importance of mouth breathing prevention.

We therefore developed a screening questionnaire for identifying mouth breathers on a numerical scale, using scores representing the number of factors characteristic of mouth breathing. We also developed, as an additional useful tool for diagnosis, a list of items for visual assessment by dental professionals (including a simple respiratory test), and combined them with the screening questionnaire into a sample screening form for habitual mouth breathing.

## Methods

To create the questionnaire, we carried out two surveys of dental patients. In both surveys, researchers explained to the patients the aim of the study and its privacy policy, and the patient’s right to refuse to participate. Only those patients giving their informed consent were asked to complete the questionnaire and submit to a visual orofacial evaluation. Informed consent was obtained in accordance with the procedures approved by the KatoBrain Co. Ltd. Research Ethics Committee, based on the Declaration of Helsinki.

### Part 1

#### Creation of the first questionnaire and oral examination points

A draft questionnaire was created consisting of 50 questions, in which the respondents were asked about a variety of items conventionally reported to be characteristic of habitual mouth breathing [19]. Of the 50 questions, 27 had 3 possible responses (yes, sometimes, or no), and 23 had 2 possible responses (yes or no). Additional questions intended to reveal possible attributes of the mouth breathing group were also included, concerning gender, age, height, weight, history of asthma, incidence of allergic rhinitis, and history of smoking.

In addition, an intraoral and orofacial examination was performed by a dental professional to visually assess the condition and morphology of the oral cavity. The examination included the following items: open bite, maxillary protrusion, mandibular protrusion (including anterior cross bite), habitual tongue thrust, chin muscle tone during lip closure, excessive overbite (overjet), other malocclusion (crowded teeth, edge-to-edge bite, cross bite), and open mouth at rest.

#### Procedures

The participants were dental clinic patients in 4 dental clinics in different cities in Japan. They were selected at random with respect to their main complaints. After a participant completed the questionnaire, a dentist or a dental hygienist under the direction of a dentist performed an oral evaluation. Dental examiners all belong to the same study group and share similar diagnostic/judgment criteria and accuracy. The oral evaluation was performed with the participant in the dental chair. Oral evaluation consisted of checking the dental chart, and inspecting for occlusion, lip closure at rest, and abnormal swallowing (tongue thrust).

Following the above procedures, responses were obtained from 102 dental patients. One response (1.0%) was excluded because of omissions in the oral evaluation. There were thus 101 valid responses, for an effective response rate of 99.0%. Of the 101 participants, 43 were males and 58 were females, average age 43.3 ± 19.7 years. Seventy-eight participants were evaluated by a dentist, and 23 by a dental hygienist.

#### Analysis

For analysis, the 101 participants were divided into a nose breathing group and a suspected mouth breathing group by 2 dentists, who separately evaluated the participants. They selected those participants they suspected of habitual mouth breathing from the results of this oral evaluation based on their clinical experience, without reference to the questionnaire or the participant’s physical profile. Participants were judged to be nose breathers if neither dentist suspected habitual mouth breathing, and they were included in the suspected mouth breathing group if they were suspected of habitual mouth breathing by either dentist. Kappa coefficients were calculated to examine the reliability of inter-rater agreement in diagnosis of suspected mouth breathing, and the resulting high kappa coefficient confirmed the diagnoses to be substantially coincident (*k* = 0.69).

The 50 questions in the questionnaire were scored as follows: for the 3-choice questions, 2 points for *Yes*, 1 point for *Sometimes*, and 0 points for *No*; and for the two-choice questions, 1 point for *Yes*, and 0 points for *No*. *Yes* and *No* scoring was reversed in any negative questions. The total range of possible scores was 0–77, with higher scores indicating a stronger tendency toward mouth breathing. Questions with significantly different scores for the nose breathing group and the suspected mouth breathing group were extracted by comparing the scores for each question between the groups using an independent t-test. The significance level was 5%. Total scores were compared between groups in the same way. Sensitivity and specificity, and false-negative and false-positive rates were calculated for each question.

Quantitative data, such as height, weight and body mass index (BMI), were compared between the groups using independent t-test. The relevance of qualitative factors such as medical history and the shape of the oral cavity to habitual mouth breathing was examined by calculating odds ratios of mouth breathing for each of these factors.

### Part 2

#### Creation of the second questionnaire and oral examination points

A draft self-report questionnaire comprising 12 questions was created based on the results of the analysis of Part 1 (see below, Results). Questions 1–11 were questions that showed significant differences in response between the groups. They were all 3-choice questions, with possible responses of *Yes* (frequently), *Sometimes* (occasionally), and *No* (not at all). The question regarding mandibular protrusion was changed by the addition of illustrations, because it seemed possible that the term “mandibular protrusion” was not easily understood by the participants. Question 12, regarding childhood asthma, was added to the questionnaire from the 6 clinical items detected by odds-ratio analysis because it is easily self-reported. The remaining orofacial development items were not added to the questionnaire because they were included in the oral examination part of the form, as in Part 1.

In the oral examination, a new item was added: “Discomfort while breathing with the lips closed”. This addition was based on previous findings of the authors that brain activity changed significantly in the 30 s after the respiratory route was changed [7]. We therefore determined for this item whether the participants felt any discomfort when breathing through the nose with the mouth completely closed for 30 s. This observation of the response when the mouth breathing route is closed is equivalent to a lip seal test, which is frequently used in the diagnosis of mouth breathing [17].

#### Procedures

The participants in Part 2 of the study were also dental clinic patients, selected randomly with respect to their main dental complaint. They were asked to complete the questionnaire and undergo an oral evaluation in the same way as in Part 1 of the study. This research was carried out in the 4 dental clinics that participated in Part 1 and in one additional clinic.

Based on the above procedures, responses were obtained from 246 participants. Four were excluded because of omissions in the oral evaluation (1.6%), for an effective response rate of 98.4%. There were thus 242 participants in Part 2: 99 males, 143 females, average age 43.4 ± 19.3 years.

The clinician performing the oral evaluation determined the breathing pattern of the patient (i.e., nasal breathing, suspected mouth breathing, mouth breathing) at the time of examination. This was based only on the results of visual assessment; the oral evaluator was not shown the completed questionnaire. The oral cavity was evaluated by a dentist for 133 participants (55.0%), and by a dental hygienist for 96 participants (39.7%). Information about the oral evaluator was not provided for the remaining 13 participants (5.4%), and it is not known whether they were evaluated by a dentist or a dental hygienist.

#### Analysis of the questionnaire based on clinical evaluation

As in Part 2, the participants were divided into three groups based on clinician evaluation: a nose breathing group, a suspected mouth breathing group, and a mouth breathing group. After they completed the second questionnaire, their total HMB (habitual mouth breathing) scores were calculated: 2 points for *Yes*, 1 point for *Sometimes*, and 0 points for *No*. The possible range of total HMB scores was 0–24, with higher scores indicating a stronger tendency to mouth breathing. Sensitivity and specificity were calculated for the 12 questions of the second questionnaire, as in Part 1. Based on those results, 10 of the 12 questions were selected for the final questionnaire, and final HMB scores were calculated using the data from the initial questionnaire created in Part 2.

Using the final HMB scores, accuracy of discrimination thresholds was evaluated by applying receiver operating characteristics (ROC) analysis, and the area under the ROC curve (AUC), sensitivity and specificity were calculated. Cut-off values were further examined using the Youden index. The cut-off values dividing the nose breathing group from the suspected mouth breathing group and the nose breathing group from the mouth breathing group were then calculated.

Quantitative data such as the BMI were also compared between the groups for possible group profile information, using the independent t-test.

#### Analysis of oral examination findings and comparison with HMB scores

For the oral examination findings, odds ratios were calculated to examine associations between each item examined and mouth breathing. The suspected mouth breathing group and the mouth breathing group were taken as a single group for calculating these odds ratios, and they were compared with those of the nose breathing group.

Then, using the cut-off HMB score values determined above, the participants were classified into a mouth breathing negative group, a suspected mouth breathing group, and a mouth breathing positive group. Rates of applicability and overlap of the important clinical findings associated with mouth breathing were then calculated for each of these groups.

### Development of a final screening form

The 10-question questionnaire and the key oral evaluation items derived above were then combined into a sample final screening form.

## Results

### Part 1: First questionnaire and oral findings

#### Group attributes

Of the 101 Part 1 participants, 73 were classified into the nose breathing group (72.3%; 29 males, 44 females), and 28 into the mouth breathing group (27.7%; 14 males, 14 females). There were no significant differences between the groups in average age (nose breathing group: 44.7 ± 19.0 years; mouth breathing group: 39.5 ± 21.0 years) or BMI (nose breathing group: 21.3 ± 3.8 kg/m^2^, mouth breathing group: 21.4 ± 3.3 kg/m^2^).

#### Analysis of responses by group

Table 1 shows the 50 questions used in the first part of the study. Comparison between the groups of scores for each question showed significant score differences between the mouth breathing group and the nose breathing group for only 11 of the 50 questions. All 11 of these questions showed significantly higher scores in the mouth breathing group than in the nose breathing group (*p* < 0.05). Sensitivity of these 11 questions was 37.5–75.0%, and specificity was 73.7–88.9%, indicating high specificity for all 11 questions.

**Table 1:** Scores, sensitivity, and specificity of Part 1 questions

	Average HMB scores		*P*-value	Sensitivity (%)	Specificity (%)
	Mouth breathing group	Nose breathing group			
Do you normally breathe through your mouth?	1.3	0.7	0.001^a^	38.10	88.90
Are you concerned that you may have bad breath?	0.9	0.5	0.028^a^	40.40	84.90
As a baby, were you breast-fed? [negative question]	0.9	0.3	0.002^a^	44.70	83.90
Do you think you have an excessive overbite?	0.6	0.3	0.001^a^	48.60	83.90
Do you often have nasal congestion?	0.8	0.4	0.015^a^	37.50	81.10
Are your front teeth easily discolored?	0.6	0.4	0.045^a^	38.60	80.00
Are the gums of your front teeth often red and swollen?	0.5	0.1	0.005^a^	61.10	79.70
Is your mouth normally open?	0.7	0.3	0.016^a^	40.50	79.40
As a child (until around age 10), did you often have nasal congestion, from rhinitis or allergies?	0.8	0.4	.009^a^	42.90	79.40
When you swallow, does your tongue protrude between your front teeth?	0.1	0	.025^a^	66.70	75.30
Does your lower jaw extend beyond your upper jaw?	0.1	0	.034^a^	75.00	73.70
Are you a fast eater?	1.1	0.9	0.34	33.30	80.50
Does your mouth feel sticky when you wake up?	1.1	0.7	0.05	32.30	79.50
Do you snore when sleeping? (Or, have you been told that you snore?	1	0.8	0.375	30.60	78.90
Is your mouth and/or throat dry and/or sore when you wake up?	0.7	0.4	0.053	33.30	78.20
How many times a day do you brush your teeth?	0.8	0.7	0.309	31.40	78.10
Does the tip of your tongue normally not touch anywhere in your mouth?	0.4	0.2	0.176	45.00	77.50
Do you drink a lot of water during a meal?	1.1	0.8	0.17	31.70	77.50
Are you prone to tartar, even though you brush your teeth?	1	0.9	0.494	28.60	76.90
Upon waking, do you have phlegm in your throat?	0.5	0.3	0.067	37.90	76.40
Can you curl your tongue, as in this photo?	0.4	0.2	0.225	35.70	76.40
Are you more comfortable breathing through your mouth?	0.3	0.2	0.108	42.10	76.30
Is your lower jaw small? (Or, have you been told that it is?)	0.4	0.2	0.066	43.50	76.30
Are you overweight?	0.3	0.2	0.201	38.10	75.90
Are you prone to cavities on your front teeth?	0.3	0.2	0.168	39.10	75.60
Do you normally chew on only one side of your mouth?	0.5	0.4	0.472	31.10	75.50
Do you catch cold easily?	0.3	0.2	0.131	42.10	75.30
Is it hard for you to bite off food with your front teeth?	0.4	0.5	0.891	26.90	75.00
Do you have poor occlusion, or are your teeth not properly aligned? (Or, have you been told so?)	0.5	0.5	0.451	31.90	75.00
Can you wiggle your nose?	0.2	0.2	0.929	26.30	74.70
Do you have difficulty breathing (nasal congestion) when you are lying down?	0.1	0	0.479	40.00	74.50%
Do you often have chapped lips or dry mouth?	0.8	0.7	0.285	29.30	74.40%
Do the corners of your mouth turn down?	0.3	0.2	0.159	42.10	74.40%
Do you have a history of smoking?	0.4	0.3	0.449	33.30	74.20%
Are you able to swallow saliva with your mouth open?	0.5	0.4	0.882	27.30	74.10%
Do wrinkles appear on your chin when you close your lips?	0.3	0.2	0.508	33.30	74.00%
Is the tip of your tongue touching your front teeth (or just behind your front teeth) right now?	0.7	0.5	0.352	32.30	73.90
Can you whistle?	0.3	0.3	0.75	29.60	73.60
Do you make chewing noises when you eat?	0.2	0.2	0.606	35.30	73.50
Have you ever had orthodontic treatment?	0.2	0.1	0.51	35.70	72.90
Do you exercise to the point of shortness of breath?	0.7	0.6	0.623	29.30	72.70
Have you had a tooth or teeth pulled for orthodontic reasons?	0.1	0.1	0.7	33.30	72.10
Does food often spill from your mouth?	0.1	0.1	0.825	25.00	71.90
Do your gums bleed?	0.4	0.5	0.846	25.60	71.70
Is the thickness of your upper and lower lips markedly different?	0	0	0.883	25.00	71.60
Do you chew your food well when you eat?	1.1	1	0.618	28.20	71.40
Do you grind your teeth in your sleep? (Or, have you been told that you do?)	0.4	0.5	0.347	25.00	70.60
Can you chew even hard food well?	0.1	0.2	0.314	17.60	70.20
Do you often have sores in your mouth?	0.5	0.7	0.198	22.90	67.90
Do you normally sleep on your side, or on your stomach?	1.1	1.3	0.226	25.00	58.80

^a^Significance

#### Analysis of medical history and orofacial examination items

Table 2 shows the odds ratios for the medical history and oral examination items. Of the medical history items, only asthma (under age 16) showed a significantly higher odds ratio. Of the oral examination items, 5 items related to orofacial development showed significantly higher odds ratios: open mouth at rest, tongue thrust during swallowing, maxillary protrusion, increased chin muscle tone with lips closed, and mandibular protrusion.

**Table 2:** Odds ratio analysis of medical history and orofacial development items (Part 1)

		Percent applicable (%)		Odds ratio
		Nose breathing group	Mouth breathing group	
Medical history	Asthma (before age 16)	4.1	28.6	9.33^a^
	Asthma (age 16 or older)	2.7	10.7	4.26
	Allergic rhinitis	4.1	10.7	2.80
	Atopy (allergies)	2.7	7.1	2.73
	History of smoking	28.8	35.7	1.38
Orofacial development	Open mouth at rest	1.4	21.4	19.64^a^
	Tongue thrust during swallowing	4.1	42.9	17.50^a^
	Maxillary protrusion	2.7	28.6	14.20^a^
	Increased chin muscle tonus	1.4	14.3	12.00^a^
	Mandibular protrusion	4.1	17.9	5.07^a^
	Crowding	23.3	28.6	1.32
	Edge-to-edge bite	6.8	3.6	0.5
	Excessive overbite	1.4	0	0
	Underbite	0	3.6	–
	Open bite	0	7.1	–

^a^Significance

### Part 2: Second questionnaire and oral findings

#### Group attributes

Breathing styles of the 242 participants were classified by the examining dentist or dental hygienist into a nose-breathing group (72.7%; (*N* = 176; 67 males, 109 females), a suspected mouth-breathing group (16.5%; *N* = 40; 18 males, 22 females), and a mouth-breathing group (10.7%; *N* = 26; 14 males, 12 females). There were no significant differences in average age or BMI between the groups: average ages for the nose breathing group, the suspected mouth breathing group, and the mouth breathing group were 44.2 ± 19.2 years, 42.9 ± 16.8 years, and 39.5 ± 23.2 years, respectively. Average BMI values for the nose breathing group, the suspected mouth breathing group, and the mouth breathing group were 21.7 ± 3.4 kg/m^2^, 21.8 ± 4.3 kg/m^2^, and 20.8 ± 3.3 kg/m^2^, respectively.

#### Analysis of the questionnaire

Table 3 shows sensitivity and specificity for each of the 12 questions for the nose and mouth breathing groups. Sensitivity exceeded 70% for 4 questions (questions 1, 2, 3 and 6). Specificity exceeded 70% for 8 questions (questions 2, 3, 4, 7, 8, 9, 11 and 12). Sensitivity for 3 questions was particularly low (questions 8, 9 and 12).

**Table 3:** Sensitivity and specificity of the screening questionnaire, second draft (Part 2)

		Sensitivity (%)	Specificity (%)
1. Do you often have nasal congestion?		73.1	69.9
2. Is your mouth normally open?		80.8	78.4
3. Do you normally breathe through your mouth?		84.0	70.7
4. Are the gums of your front teeth often red and swollen?		42.3	80.7
5. Are your front teeth easily discolored?		65.4	63.1
6. Are you concerned that you may have bad breath?		76.9	52.8
7. Do you think you have an excessive overbite?		42.3	76.7
8. When you bite naturally, does your lower jaw extend excessively (mandibular protrusion)? Answer as follows: Yes, if your lower teeth extend beyond upper teeth, as in Fig. a Sometimes, if your upper and lower teeth are aligned, edge to edge, as in Fig. b No, if your upper teeth extend over your lower teeth		23.1	83.8
(A)	(B)		
9. When you swallow, does your tongue protrude between your teeth?		30.8	93.1
10. As an infant, were you bottle-fed, breast-fed, or both? Answer as follows:Yes, if bottle-fed onlySometimes, if bothNo, if breast-fed only		50.0	63.6
11. As a child (until around age 10), did you often have nasal congestion, from rhinitis or allergies?		60.0	72.0
12. Before age 16, were you ever diagnosed with bronchial asthma? Answer as follows: Yes, if you had asthma attacks after age 16 Sometimes, if you had asthma before age 16, but not afterwards. No, if you were never diagnosed with asthma.		17.4	92.3

#### Creation of the final questionnaire

We then examined the questions from Table 3 with low sensitivity and a high false negative rate (Q8, Q9 and Q12) individually to determine whether they should be included in the final screening form.

Question 8 concerned mandibular protrusion and edge-to-edge bite. Its sensitivity was low, and the odds ratios for mandibular protrusion and edge-to-edge bite, indicating anterior cross bite, were not significant. For these reasons, it was considered a low-precision item, whether self-reported or observed in oral examination, and so excluded.

Question 9 concerned tongue thrust during swallowing. The significant odds ratio indicates that tongue thrust during swallowing is a characteristic oral finding in the mouth breathing group. However, its sensitivity was low, showing that participants were likely to be unaware of it. It was therefore excluded, because it could be more effectively observed by a dental professional.

The odds ratio of question 12 (history of asthma) was highly significant, suggesting that a history of asthma is strongly relevant to mouth breathing. Question 12 also showed low sensitivity, but its specificity was high (92.3%). We therefore decided to include Question 12 in the final questionnaire, because a history of asthma is easily self-reported.

After excluding questions 8 and 9, we created a final screening questionnaire of 10 questions.

#### Evaluation of the final questionnaire

HMB scores calculated using the data from the initial questionnaire created in Part 2, aggregating only the answers of those questions included in the final draft screening questionnaire.

Average total screening HMB scores were 3.5 ± 2.6 for the nose breathing group, 5.8 ± 3.3 for the suspected mouth breathing group, and 8.7 ± 4.5 for the mouth breathing group. The difference in average total scores between the nose breathing group and the suspected mouth breathing group (*p* = 0.466) was not significant. The average total score of the mouth breathing group was significantly higher than the scores of the nose breathing group and the suspected mouth breathing group (*p* < 0.000).

**Fig. 1:**
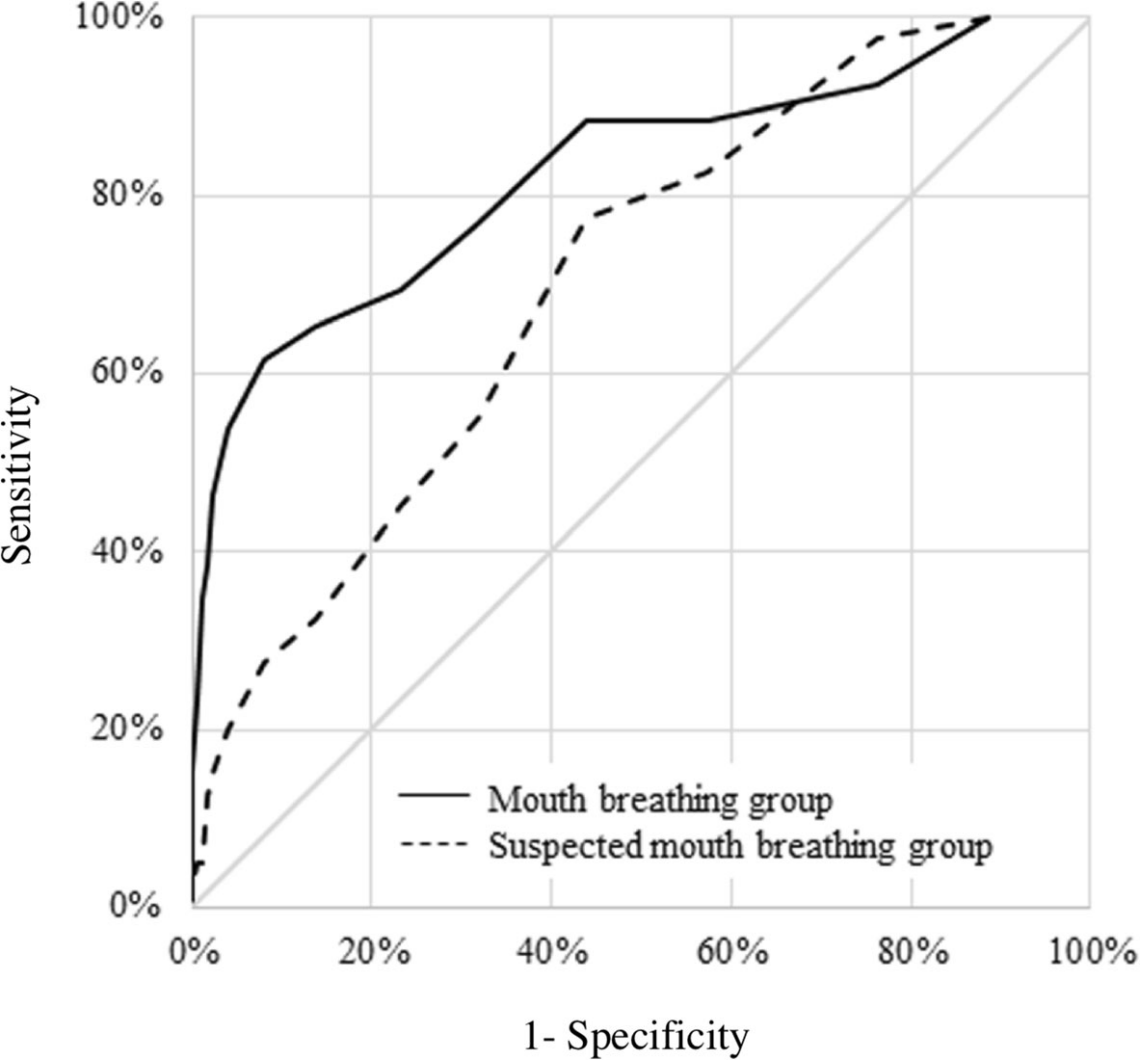
ROC curve (solid line: mouth breathing group; dotted line: suspected mouth breathing group)

ROC curves (Fig. 1) and AUC were calculated to confirm accuracy of diagnosis. AUC were 0.825 for the mouth breathing group, and 0.705 for the suspected mouth breathing group. These values indicate moderate diagnostic performance for the final screening questionnaire.

Youden indices were calculated to determine HMB score cut-off values. In the case of the nose breathing group and the mouth breathing group, the Youden index was highest when the cut-off value was 8 points (Youden index: 0.536; sensitivity: 61.5%; specificity: 92.0%). In the case of the nose-breathing and suspected mouth-breathing groups, the Youden index was highest when the cut-off value was 4 points (Youden index: 0.338; sensitivity: 77.5%; specificity: 56.3%).

The above results suggest that it is possible to diagnose a high likelihood of habitual nose breathing from a score of 0–3, a suspicion of mouth breathing from a score of 4–7, and a high likelihood of habitual mouth breathing from a score of 8 points or higher.

When the mouth breathing and suspected mouth breathing groups were combined, scores of ≥4 showed 81.8% specificity and 56.3% sensitivity, and scores of ≥8 showed 40.9% sensitivity and 92.0% specificity.

#### Analysis of medical history and orofacial examination items

Odds ratios calculated for the results of the oral examination (Table 4) showed 6 items with significantly high odds ratios for the combined mouth breathing group (mouth-breathing and suspected mouth-breathing groups) vs. the nose breathing group. Of these 6 items, the item with the highest odds ratio was discomfort while breathing with the lips closed, followed in order by open mouth at rest, increased chin muscle tone with lips closed, tongue thrust during swallowing, maxillary protrusion, and open bite. Odds ratios were not significant for mandibular protrusion, edge-to-edge bite, other malocclusion, and excessive overbite. The odds ratio for history of asthma, calculated from the self-report questionnaire responses, was also significantly higher in the combined mouth-breathing / suspected mouth-breathing groups.

**Table 4:** Odds ratio analysis of oral examination items (Part 2)

	Applicability (%)		Odds ratio
	Nose breathing group	Mouth breathing group (includes suspected mouth breathing group)	
Discomfort while breathing with the lips closed	1.1	36.4	49.71^a^
Open mouth at rest	4.5	63.6	36.75^a^
Increased chin muscle tonus	3.4	19.7	6.95^a^
Tongue thrust during swallowing	9.7	42.4	6.89^a^
Maxillary protrusion	8.0	22.7	3.40^a^
Open bite	4.5	12.1	2.90^a^
History of asthma	7.4	16.7	2.51^a^
Mandibular protrusion	6.8	15.2	2.44
Edge-to-edge bite	1.7	3.0	1.80
Other malocclusion (crowding, cross bite)	17.6	27.3	1.50
Excessive overbite	2.3	0	0.00

^a^Significance

These results suggest that the seven items of discomfort while breathing with the lips closed, open mouth at rest, increased chin muscle tone with lips closed, tongue thrust during swallowing, maxillary protrusion, open bite, and history of asthma are important indicators of a tendency to mouth breathing.

#### Correspondence between HMB scores and 7 key clinical items

HMB scores from the final questionnaire showed 45.9% of the participants (*N* = 111) to be mouth-breathing negative (HMB score 0–3), 37.2% of the participants (*N* = 90) to be suspected mouth breathers (HMB score 4–7), and 16.9% of participants (*N* = 41) to be mouth breathing positive (HMB score ≥ 8). Average HMB scores were 1.6 ± 1.0 for the mouth-breathing negative group (nose-breathing group), 5.2 ± 1.1 for the suspected mouth-breathing group, and 10.4 ± 2.3 for the mouth-breathing positive group.

Table 5 shows rates of applicability for each group of the 7 key clinical items in the questionnaire. For all 7 items, applicability was highest for the mouth breathing positive group, followed in order by the suspected mouth breathing group and then the mouth breathing negative group.

**Table 5:** Applicability of oral examination items by group (classified according to HMB scores)

	Percent applicability (%)		
	Mouth breathing negative group	Suspected mouth breathing group	Mouth breathing positive group
Discomfort while breathing with the lips closed	2.7	12.2	29.3
Open mouth at rest	9.9	17.8	56.1
Increased chin muscle tonus	3.6	10.0	14.6
Tongue thrust during swallowing	10.8	23.3	29.3
Maxillary protrusion	8.1	10.0	26.8
Open bite	3.6	8.9	9.8
History of asthma	1.8	10.0	31.7

**Fig. 2:**
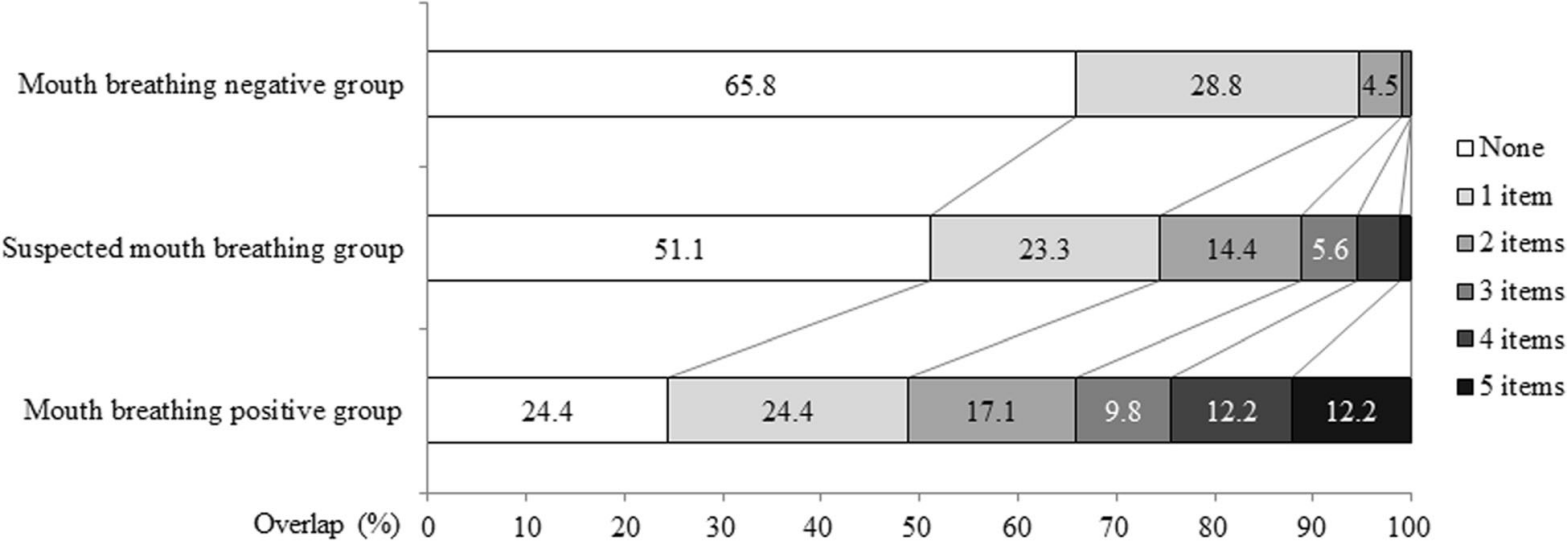
Overlap of oral examination items by group (classified by HMB scores)

Results of calculating overlap for the seven key clinical items (Fig. 2) showed no relevant items for 65.8% of the mouth breathing negative group, and only relevant 1 item for 28.8% of this group; in other words, no more than 1 item applied to 95% of the mouth breathing negative group. Applicability was approximately 50% for the suspected mouth breathing group, with 1–2 items applying to approximately 40% of this group, and 3–5 items applying to the remaining 10%. None of the items applied to 25% of the mouth breathing positive group, 1 item applied to another 25% of this group, and 2–5 items applied to the remaining 50%.

## Discussion

### Contents of the sample screening form

In this study, we created a sample habitual mouth breathing screening form, consisting of a 10-question patient questionnaire and a 6-item oral examination. Cut-off values for the resulting HMB scores from the questionnaire were determined for diagnosis. Scores of 0–3 (out of 20) were found to indicate a high likelihood of habitual nose breathing; scores of 4–7 indicated a suspicion of mouth breathing; and scores of 8 or more indicated a high likelihood of habitual mouth breathing. HMB screening cannot completely differentiate habitual mouth breathers and nose breathers, but it is characterized by its use of an incremental scale to evaluate a respondent as a mouth breather, a suspected mouth breather, or a nose breather. A diagnosis of suspected mouth breathing makes possible further evaluation and possibly intervention from the standpoint of preventing habitual mouth breathing.

The questions we found to be effective in differentiating nose and mouth breathing were 10 questions regarding chronic nasal congestion, chronic open mouth (open mouth posture), the respondent’s awareness of habitual mouth breathing (outside of exercise), gum swelling, staining of the front teeth, bad breath, excessive overbite (mandibular protrusion), chronic nasal congestion in childhood, whether the respondent was bottle-fed or breast-fed as an infant, and a history of childhood asthma.

Eight of these items are consistent with findings previously reported to be characteristic of habitual mouth breathing [13]. Chronic nasal congestion and chronic open mouth are both related to the respiratory route, and obstruction of the nasal breathing route or leaving the mouth open over long periods of time while at rest has been shown to result in a tendency towards habitual mouth breathing. The question regarding self-awareness of mouth breathing is a question with higher sensitivity and specificity, and was suggested as one that could be easily answered by adult respondents, as in the present study. Dry mouth is a change in the oral environment likely be caused by habitual mouth breathing; and gum swelling at the front teeth [20, 21], staining of the front teeth [19], and bad breath [22] are found associated with dry mouth; we thus considered them to be valid questions. The question concerning mandibular protrusion / excessive overbite (overjet) concerns the shape of the oral cavity and can be considered to include labial inclination of the maxillary frontal teeth, maxillary protrusion and mandibular retrusion [20, 23–27]. Excessive overbite makes it difficult to close the mouth breathing route, and its association with chronic open mouth suggests the possibility of habitual mouth breathing. Whether a respondent was bottle-fed or breast-fed as an infant is related to growth history, and our finding is not inconsistent with previous studies showing bottle-feeding to be associated with a tendency to mouth-breathing [28, 29]. Breast feeding requires more work for an infant than bottle feeding, and has been reported to encourage development of muscle function around the mouth as well as stimulation of the breathing, swallowing and chewing functions [30]. It can thus be considered to be important in the acquisition of normal oral function.

Two questions newly extracted in this study are related to growth history: chronic nasal congestion in childhood and a history of childhood asthma. Childhood chronic nasal congestion, like chronic nasal congestion as an adult, blocks off the nasal passages and can thus result in habitual closing of the nasal respiratory route. In asthma, mouth breathing during asthma attacks can lead to habitual mouth breathing when the mouth breathing route is not closed off after the attack. The present study, which only included adults, suggests that breathing habits in acquired in childhood may result in habitual mouth breathing in adulthood. Previous research has advocated the importance of proper treatment of childhood habitual mouth breathing in preventing the continuation of mouth breathing patterns into adulthood [17]. In the present study, it was impossible to confirm the childhood breathing habits of the participants, and clarification of a direct relationship will require further study. However, the importance suggested here of asking adults about their developmental history is interesting.

We thus considered elements of orofacial morphology, such as maxillary protrusion, chronic closure of the nasal breathing route, and use of the mouth breathing route when breathing is difficult, as risk factors for habitual mouth breathing. In other words, habitual mouth breathing can be considered likely to result from erroneous learning or inevitable adaptation of the respiratory route in the developmental process. This suggests that educational intervention related to breathing is important over the long term for high-risk children.

On the other hand, regarding other conventional findings such as snoring [19] and dental caries [31], we did not find questions about these characteristics to be statistically valid. Snoring is considered to indicate habitual mouth breathing, but only during sleep. A person is unlikely to be consciously aware of snoring, and we believe that this is why snoring was not detected to a significant extent in this study. Because of its association with snoring, the BMI of each participant was calculated in order to investigate a possible relationship between obesity and mouth breathing [32]. The BMIs of all participants, however, were within the normal weight range, with no significant difference between the groups; that is, we found no direct relationship between weight and habitual mouth breathing. Questions regarding dental findings, such as dental caries, were also statistically rejected. General dental findings are not observed only in habitual mouth breathers; they are also observed in (and reported by) nose breathers. This also means that erroneous diagnosis may result if diagnosis is based on dental findings conventionally thought to be related to mouth breathing without other verification.

### Clinical oral findings associated with mouth breathing

Odds ratio analysis was performed to show which oral findings were likely to be associated with habitual mouth breathing. Excluding the questionnaire item regarding asthma, odds analysis revealed 6 items likely to accompany mouth breathing: discomfort while breathing with lips closed, open mouth at rest, increased chin-muscle tone with lips closed, tongue thrust during swallowing, maxillary protrusion, and open bite. Among these, significant odds ratio were reproducibly detected in both survey populations for 4 items. The two items discomfort while breathing with lips closed and open bite were not included in the first survey.

The items open mouth at rest and maxillary protrusion were included in both the questionnaire and the oral examination, and had a high rate of detection in both clinical oral evaluation and self-reporting. In contrast, the items increased chin muscle tone with lips closed, tongue thrust during swallowing, open bite, and malocclusion were rejected in the self-report questionnaire but were statistically validated in the clinical evaluation. This led us to believe that subjective answers self-reported by the respondents are to some extent effective, but the inclusion of a clinical examination by a health professional is also necessary for a final diagnosis of mouth breathing.

With respect to discomfort while breathing with lips closed, the brain oxygen exchange load has been found to be increased when breathing through the non-habitual respiratory route, especially during the first 30 s [7]. In the future, it is conceivable that measurement of brain function might be performed on dental patients with high HMB scores, to physiologically observe effects of mouth vs. nose breathing.

Using HMB scores for diagnosis, we found that 0–1 of the 7 key clinical items applied to approximately 95% of mouth breathing negative respondents. For suspected mouth breathers, 1–5 items applied to approximately 50% of respondents. For mouth breathing positive respondents, 1 or more item applied to approximately 75% of respondents, and 2 or more applied to approximately 50% of respondents. Particularly for those respondents with HMB scores of 4–7 (suspected mouth breathers), overall clinical evaluation based on the presence of clinical findings reported in the final screening form is likely to increase the accuracy of screening.

### Importance of screening for habitual mouth breathing

Our results are based on data from a total of approximately 350 participants in parts 1 and 2 of this study. The percentages of mouth breathers were 27.7% for the first questionnaire, and 10.1% for the second. Previous studies of adults have reported 13–26% of total subjects to be mouth breathers [13, 33]. In our study we observed a discrepancy in the percentages of mouth breathers, with the first questionnaires identifying a higher number of suspected mouth breathers compared to the second. We believe this is due to increased accuracy with the specialized questionnaire in the second group.

When one chews while breathing through the mouth, jaw movement and chewing efficiency are reduced by resulting factors such as changes in moisture content and breathing cycles [34, 35]. It has been reported that for people who normally have difficulty keeping their lips closed, it is difficult to chew with the mouth closed, and this can have a harmful effect on masticatory function [36].

Despite this and other harmful effects of mouth breathing, habitual mouth breathing is not sufficiently recognized as a habit requiring intervention. Habitual mouth breathers themselves may also be unaware of this habit. Against this background, one is unlikely to visit a dentist specifically to ask about mouth breathing, and intervention may be delayed. In addition, treatment of childhood asthma, which is shown in this study to be a factor in habitual mouth breathing, consists mainly of bringing the attacks under control, and it does not normally involve active intervention to prevent possible subsequent changes in breathing habits.

Effective use of screening would make it possible to ascertain the breathing habits of dental patients who visit the dentist for other reasons. It could lead to the implementation of comprehensive oral care that takes into account the causal relationship between mouth breathing and other complaints. Furthermore, use of a screening questionnaire could promote general awareness of habitual mouth breathing as well as objective diagnosis. Particularly in the case of persons who are unaware of their habitual mouth breathing, this could then facilitate cooperation in intervention. A screening questionnaire could moreover be made available on the internet, further helping to prevent unawareness of habitual mouth breathing by increasing awareness of mouth breathing as a problem, and encouraging consultations regarding habitual mouth breathing.

Effective screening questionnaires for medical problems such as sleep apnea [37], attention deficit disorder [38], and asthma [39] are already well established in the field of medicine. In the field of dentistry, however, there has been no such statistically established screening. A screening form for habitual mouth breathing could help in establishing standard diagnostic criteria for habitual mouth breathing. The finding in this study of an intermediate group, who are only suspected of habitual mouth breathing, shows that there are patients who are not easy to diagnose. The use of a variety of different diagnostic criteria for mouth breathing increases the risk of missed diagnoses at this stage. Screening using HMB scores could be useful in possibly increasing the consensus among dentists regarding diagnostic criteria for mouth breathing, as well as quantitatively identifying a tendency towards mouth breathing. Furthermore, because screening of this kind can be completed easily and quickly, it could also be used to identify persons with possible habitual mouth breathing in situations like regularly scheduled medical checkups.

Determination of nasal and mouth breathing modes in the present study was carried out from a clinical dental point of view and was based on visual evaluation results of patients in a dental care setting. There are previous studies that have been skeptical of the possibility of determining breathing modes by visual assessment or interview alone. One report states the need to measure air flow from the mouth and nose using electrical devices to determine the breathing mode [11]. We believe that it will be important to investigate the relationship between the HMB scores developed clinically in this study and breathing modes measured in this way, in order to assess the validity of the screening questionnaire created in this study.

## Conclusion

We propose in this study a systematized screening questionnaire for detecting habitual mouth breathing. Changes in HMB scores can possibly be used to track and verify the effectiveness of the treatment to break the habit of mouth breathing. A standardized HMB screening process could be a useful contribution to evidence-based dental medicine. Moreover, this study suggests that a history of breast-feeding and nasal congestion and/or asthma in childhood is likely to be involved in the development of mouth breathing. Future studies focusing on the direct relationship between developmental histories and breathing habits may result in findings that will help prevent the development of habitual mouth breathing. This study used a convenience sample drawn from patients with dental problems. Future studies should assess the validity and accuracy of the proposed index in other populations of patients with dental problems as well as in non-patient populations.

## Abbreviations

AUC: Area under the ROC curve
BMI: Body mass index
HMB: Habitual mouth breathing
ROC: Receiver operating characteristics
